# Etiology and risk factors of ischemic stroke during pregnancy and puerperium: A population-based study

**DOI:** 10.1177/23969873231170096

**Published:** 2023-04-24

**Authors:** Anna Richardt, Karoliina Aarnio, Aino Korhonen, Kirsi Rantanen, Liisa Verho, Hannele Laivuori, Mika Gissler, Minna Tikkanen, Petra Ijäs

**Affiliations:** 1 Department of Neurology, University of Helsinki and Helsinki University Hospital, Helsinki, Finland; 2 Department of Obstetrics and Gynecology, University of Helsinki and Helsinki University Hospital, Helsinki, Finland; 3 Department of Medical and Clinical Genetics, University of Helsinki and Helsinki University Hospital, Helsinki, Finland; 4 Institute for Molecular Medicine Finland, Helsinki Institute of Life Science, University of Helsinki, Helsinki, Finland; 5 Department of Obstetrics and Gynecology, Tampere University Hospital, Tampere, Finland; 6 Center for Child, Adolescent and Maternal Health Research, Faculty of Medicine and Health Technology, Tampere University, Tampere, Finland; 7 Department of Knowledge Brokers, Finnish Institute for Health and Welfare, Helsinki, Finland; 8 Academic Primary Health Care Centre, Region Stockholm, Stockholm, Sweden; 9 Department of Molecular Medicine and Surgery, Karolinska Institute, Stockholm, Sweden

**Keywords:** Ischemic stroke, pregnancy, puerperium, postpartum period, stroke, risk factors, etiology

## Abstract

**Introduction:**

Ischemic stroke (IS) is an uncommon, but potentially life-changing, complication of pregnancy. The aim of this study was to analyze the etiology and risk factors of pregnancy-associated IS.

**Patients and methods:**

We collected a population-based retrospective cohort of patients diagnosed with IS during pregnancy or puerperium in Finland from 1987 to 2016. These women were identified by linking the Medical Birth Register (MBR) with the Hospital Discharge Register. Three matched controls were selected from MBR for each case. The diagnosis and temporal relationship of IS to pregnancy, and clinical details were verified from patient records.

**Results:**

A total of 97 women (median age 30.7 years) were identified as having pregnancy-associated IS. The most common etiologies based on TOAST classification were cardioembolism in 13 (13.4%), other determined in 27 (27.8%) and undetermined in 55 (56.7%) patients. Fifteen patients (15.5%) had embolic strokes of undetermined sources. The most important risk factors were pre-eclampsia, eclampsia, gestational hypertension, and migraine. IS patients had more frequently traditional and pregnancy-related stroke risk factors than the controls (OR 2.38, 95% CI 1.48–3.84) and the risk of IS multiplied with the number of risk factors (4–5 risk factors: OR 14.21, 95% CI 1.12–180.48).

**Discussion and conclusion:**

Rare causes and cardioembolism were frequent etiologies for pregnancy-associated IS, but in half of the women, the etiology remained undetermined. The risk of IS increased with the number of risk factors. Surveillance and counseling of pregnant women, especially with multiple risk factors, is crucial for the prevention of pregnancy-associated IS.

## Introduction

Ischemic stroke (IS) during pregnancy and puerperium is an uncommon, but devastating, event for young women and their families. The incidence of IS is estimated to be 12.2 per 100,000 pregnancies.^1^ Our group has reported a 2.2-fold increase in the incidence of pregnancy-associated stroke in Finland from 1987 to 2016.^2^ Most ISs present during the third trimester and up to 6 weeks postpartum.^3–5^ Pregnancy-related factors, such as a physiological hypercoagulable state, pre-eclampsia, and eclampsia, are associated with an increased risk of IS and other thromboembolic events.^6^ Interestingly, pregnant stroke patients are reported to have fewer traditional risk factors for stroke (i.e. diabetes, dyslipidemia, hypertension) than nonpregnant stroke patients.^7^

The data on pregnancy- and puerperium-associated IS are limited, since it is an uncommon condition. Most of the published studies have a limited number of cases and only a few are population-based or nationwide.^8–13^ Different study settings and diagnostic procedures have been used, and some studies do not differentiate between the ISs of arterial and venous origin.^3,10,14^

More studies are needed to improve our understanding of the prevention and diagnosis of pregnancy-associated IS. We aimed to analyze the risk factors and etiology of IS during pregnancy and puerperium in a nationwide population-based cohort study in Finland covering 30 years.

## Patients and methods

This article follows the STrengthening the Reporting of OBservational studies in Epidemiology (STROBE) reporting guideline (Supplemental data).^15^

### Study design and identification of patients using national registers

**Figure 1.**
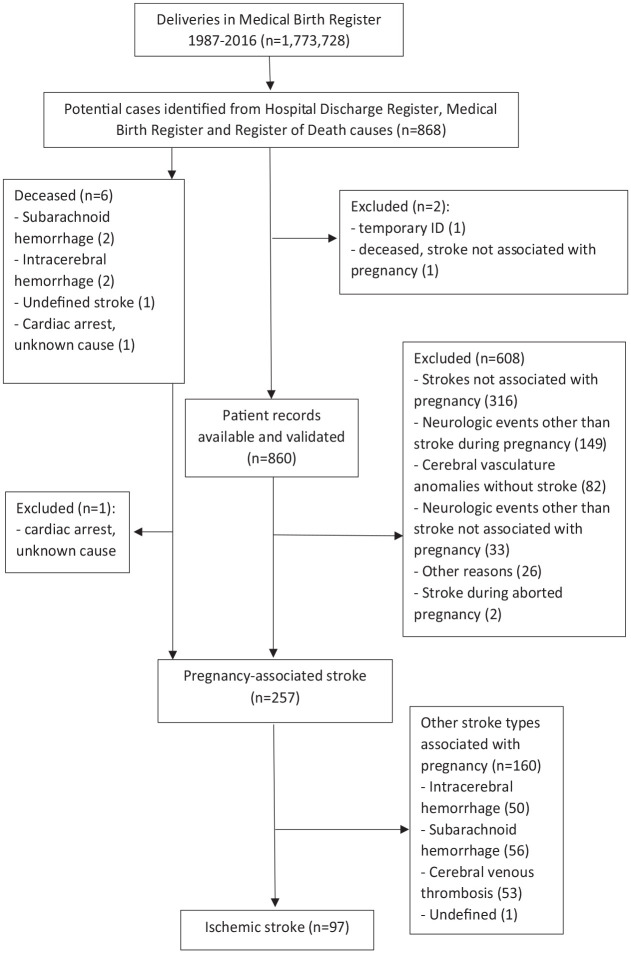
Search strategy to identify women with pregnancy-associated ischemic stroke.

We performed a population-based nationwide cohort study, and a nested case-control study. Patients diagnosed with IS during pregnancy or puerperium in Finland from 1987 to 2016 were identified by linking national healthcare registers: The Medical Birth Register (MBR), Hospital Discharge Register, and the Cause-of-Death register. The data were collected retrospectively from registers with disease or procedure codes indicating IS or its treatment in HDR up to 9 months before or up to 3 months after the delivery date in MBR. The detailed study design and search strategy are described in Figure 1 and the Supplemental Methods.

### Chart verification and definitions

Medical records of the identified patients were reviewed by our research group to verify the diagnoses, the temporal connection between stroke and pregnancy, and collect data. ISs of solely arterial origin that occurred during pregnancy or puerperium were included in the study. The diagnosis of IS and the data on risk factors, etiology, diagnostic investigations, and outcome were verified by stroke neurologists. The diagnosis of IS was based on clinical findings and radiographic features. In uncertain cases, a consensus between neurologists was reached. Detailed chart verification and definitions are described in the Supplemental Methods.

We defined the etiologies of IS using the Trial of ORG 10172 in Acute Stroke Treatment (TOAST) criteria, which included large-artery atherosclerosis, cardioembolism, small-vessel occlusion, stroke of other determined etiology, and stroke of undetermined etiology.^16^ Stroke of undetermined etiology had three subclasses: two or more causes identified, negative evaluation, or incomplete evaluation.^16^ Embolic stroke of undetermined source (ESUS) was defined as a non-lacunar stroke detected by CT or MRI, in the absence of extra- or intracranial atherosclerosis causing ⩾50% luminal stenosis, major risk cardioembolic source, and other specific cause of stroke. Further details on etiological classifications can be found in Supplemental methods. Functional outcome was estimated from medical records using the modified Rankin Scale (mRS).^17^

### Risk factor analysis of cases and controls

Three controls (pregnant women without stroke) that were matched by delivery year, age, parity, and geographic area were identified from the MBR for each IS case. No controls for one case and only one control for another case were available due to extreme age and a sparsely inhabited geographical area. Only register data from the HDR and MBR, not validated from medical records, were used in the risk-factor analysis, since medical records were not available for the controls. The data in MBR are supplemented by healthcare professionals during antenatal and postnatal (until 7 days after delivery) pregnancy surveillance visits. The HDR was searched with diagnostic codes corresponding to stroke risk factors registered during pregnancy and until 3 months after delivery (Supplemental Methods). Register data were not available for two IS cases. Cases and controls were compared using all known traditional and pregnancy-related risk factors of stroke available in the registers.

### Statistical methodology

The incidence of IS was reported per 100,000 deliveries. Confidence intervals for incidence rates were calculated based on Poisson distribution. Data are presented as numbers and percentages or median and interquartile ranges (IQR). To test for differences between the groups, χ^2^-test or Fisher’s exact test was used for dichotomous variables, *T*-test for continuous variables, and Mann-Whitney *U*-test for ordinal variables. *p*-Value <0.05 was considered statistically significant. Age-adjusted odds ratios for individual risk factors were calculated by logistic regression. For the multivariable logistic regression analysis, all variables with events in cases and controls were entered in the model. Multivariable odds ratios for cesarean section and postpartum hemorrhage were calculated only in women with postpartum IS. Statistical analyses were performed using IBM SPSS statistics, Version 27.

## Results

### Demographics and diagnostics

During 1987–2016 in Finland, 97 patients with IS during pregnancy or postpartum were identified from registers and verified from patient records (Figure 1). During this period, there were 1,773,728 deliveries, 1,792,791 live births, and 6799 stillbirths in the MBR. The median age of IS patients was 30.7 years (26.6–35.0) (Table 1). The etiology of IS per TOAST classification was undetermined in 56.7%, other determined in 27.8%, cardioembolism in 13.4%, and small-vessel occlusion in 2.1% of patients. No ISs were caused by large-artery atherosclerosis.

**Table 1.** Demographics and risk factors of ischemic stroke cases per TOAST classification.

Etiology	All	Cardioembolism	Small-vessel occlusion	Stroke of other determined etiology	Stroke of undetermined etiology
No. of cases (%)	97 (100)	13 (13.4)	2 (2.1)	27 (27.8)	55 (56.7)
Age (years), median ± IQR	30.7 ± 8.4	29.2 ± 8.9	36.7 ± 0	33.5 ± 7.4	29.4 ± 7.1
Parity, median ± IQR	2 ± 3	2 ± 4	5.5 ± 1	2 ± 3	1 ± 2
Traditional risk factors^a,b^	65 (67.0)	9 (69.2)	2 (100.0)	18 (66.7)	36 (65.5)
Obesity (BMI ⩾ 30 kg/m^2^)^a^	8 (8.2)	2 (15.4)	1 (50.0)	3 (11.1)	2 (3.6)
Smoking^b^	15 (15.5)	1 (7.7)	0	5 (18.5)	9 (16.4)
Chronic hypertension	7 (7.2)	1 (7.7)	0	3 (11.1)	3 (5.5)
Hypercholesterolemia	35 (36.1)	5 (38.5)	0	8 (29.6)	22 (40.0)
Migraine	34 (35.1)	5 (38.5)	1 (50.0)	10 (37.0)	18 (32.7)
Migraine with aura	28 (29.5)	5 (38.5)	1 (50.0)	7 (25.9)	15 (27.3)
Diabetes, type I or II	0	0	0	0	0
Pregnancy-related risk factors	34 (35.1)	3 (23.1)	1 (50.0)	15 (55.6)	15 (27.3)
Gestational diabetes	16 (16.5)	2 (15.4)	1 (50.0)	6 (22.2)	7 (12.7)
Gestational hypertension	10 (10.4)	0	0	5 (18.5)	5 (9.1)
Pre-eclampsia^c^	21 (21.6)	2 (15.4)	0	13 (48.1)	6 (10.9)
Hypertensive disorders of pregnancy^d^	28 (28.7)	2 (15.4)	0	15 (55.6)	11 (20.0)
Prior prothrombotic disorder	3 (3.1)	0	0	3 (11.1)	0
Prior cardiac disease	9 (9.3)	4 (30.8)	0	2 (7.4)	3 (5.5)
Immune system disease	3 (3.1)	0	0	2 (7.4)	1 (1.8)
Previous stroke	5 (5.2)	2 (15.4)	0	0	3 (5.5)
Family history of stroke	10 (10.3)	3 (23.1)	0	1 (3.7)	6 (10.9)

BMI: body mass index; HELLP: hemolysis, elevated liver enzymes, low platelets; IQR: interquartile range.

Data are presented as *n* (%) and median ± IQR. Data were collected from medical records.

a BMI data missing in 44 cases.

b Smoking data missing in four cases.

c Includes eclampsia and HELLP.

d Pre-eclampsia, eclampsia, HELLP, gestational hypertension, and/or chronic hypertension.

All patients underwent a brain CT, MRI, or both and 84.5% of the patients underwent vessel imaging (Supplemental Table 1). Holter monitoring was performed on 29.9% and echocardiograms on 80.4% of the patients. Laboratory examinations for the prothrombotic state were investigated in 66.0% of the patients.

Of IS patients, 67.0% had at least one traditional risk factor: 15.5% smoked cigarettes, 7.2% had chronic hypertension, 36.1% had hypercholesterolemia, 35.1% had migraine, and 8.2% were obese, but none had type 1 or 2 diabetes. Of patients, 35.1% had at least one pregnancy-related risk factor: 16.5% had gestational diabetes, 10.4% had gestational hypertension, and 21.6% had pre-eclampsia, eclampsia or hemolysis, elevated liver enzymes, low platelets syndrome (HELLP).

Maternal in-hospital mortality was 5.2% and there were no additional deaths until the end of follow-up. Three deaths occurred in patients with other determined etiologies. All had HELLP, two with associated disseminated intravascular coagulation (DIC) and one had associated posterior reversible encephalopathy syndrome (PRES). There was one death each for cardioembolic and undetermined etiology. The median mRS was 2.0 (IQR ± 2) at discharge and 1.0 (IQR ± 2) at 3 months. In total, 83.5% of the patients had good recovery (mRS 0–2) at 3 months.

### Etiology of ischemic strokes

Cardioembolisms were most commonly due to atrial septal defect (38.5%) or patent foramen ovale (30.8%). Other determined etiologies were dissection of the cervical or intracranial artery in 40.7% of cases, HELLP complicated by DIC in 11.1% cases, Factor V Leiden mutation, and anti-phospholipid syndrome in 11.1% of cases each, and hyperstimulation syndrome in 7.4% of cases. Rarer specified etiologies were hypovolemia, air embolism, moyamoya disease, PRES associated with HELLP, and migraine, accounting for 3.7% of cases each.

The diagnostic evaluation was negative in 74.6% and incomplete in 23.6% of the ISs of undetermined etiology. Of incompletely evaluated patients, 61.5% had no vessel imaging, 38.5% had no echocardiogram, and 30.7% had no electrocardiogram. Most of the cases with incomplete work-up (77.0%) occurred in 1987–1996.

Criteria for ESUS were met in 15.5% of patients.^18^ Of ISs with undetermined and negatively evaluated etiology, 24.4% had ESUS. From all the ESUSs, 26.7% presented in the first trimester, 13.3% in the second trimester, 26.7% in the third trimester, and 33.3% during postpartum.

The data for the IS cases with negative evaluation compared to other IS cases are presented in Supplemental Table 3. Patients with a negative diagnostic evaluation had less frequently hypertensive disorders of pregnancy (HDP) than did other IS patients (17.1% vs 37.5%, OR 0.34, 95% CI 0.13–0.91). Patients with a negative diagnostic evaluation had more commonly good functional outcome (mRS 0–2) at hospital discharge (85.4% vs 64.3%, OR 3.24, 95% CI 1.16–9.02).

### Risk factor analysis

A total of 95 IS cases and 280 controls were included in the risk factor analyses (Tables 2 and 3). Of the IS cases, 14.7% had pre-eclampsia or eclampsia compared to only 3.2% of the controls (OR 5.20, 95% CI 2.17–12.27). A larger proportion of IS patients had gestational hypertension (11.6% vs 4.6%, OR 2.69, 95% CI 1.16–6.23) and migraine (11.6%vs 0.4%, (OR 36.54, 95% CI 4.65–287.12) than did the controls. Cesarean section (OR 2.77, 95% CI 1.39–5.51) was a risk factor for postpartum IS.

**Table 2.** Risk factors for ischemic stroke cases and controls.

Variable	Cases	Controls	All	Unadjusted	Age-adjusted	Multivariable
	*n* = 95	*n* = 280	*n* = 375	OR (95% CI)	OR (95% CI)	OR (95% CI)
Age (years), median ± IQR	30.0 ± 9.0	30.0 ± 8.8	30.0 ± 9.0	1.00 (0.96–1.05)		0.95 (0.90–1.00)
Parity, median ± IQR	1.0 ± 2.0	1.0 ± 2.0	1.0 ± 2.0	1.19 (1.03–1.39)	1.23 (1.04–1.44)	1.35 (1.12–1.62)
Traditional risk factors						
Obesity (BMI ⩾ 30 kg/m^2^)^a^	8 (8.4)	18 (6.4)	26 (6.9)	1.38 (0.56–3.39)	(0.55–3.87)	1.60 (0.53–4.86)
Smoking^b^	15 (15.8)	40 (14.3)	55 (14.7)	1.17 (0.61–2.23)	(0.59–2.33)	0.95 (0.46–1.99)
Chronic hypertension	3 (3.2)	1 (0.4)	4 (1.1)	9.10 (0.94–88.54)	(0.93–89.99)	4.99 (0.38–66.15)
Hypercholesterolemia	3 (3.2)	2 (0.7)	5 (1.3)	4.53 (0.75–27.55)	(0.75–27.77)	0.47 (0.04–5.12)
Migraine	11 (11.6)	1 (0.4)	12 (3.2)	36.54 (4.65–287.12)	36.52 (4.65–287.00)	46.00 (5.69–372.18)
Diabetes mellitus^c^	0	1 (0.4)	1 (0.3)	N/A	N/A	N/A
Pregnancy-related risk factors						
Diabetes during pregnancy^d^	15 (15.8)	26 (9.3)	41 (10.9)	1.83 (0.93–3.63)	1.83 (0.92–3.64)	1.81 (0.82–4.03)
Gestational hypertension^e^	11 (11.6)	14 (4.6)	24 (6.4)	2.69 (1.16–6.23)	2.71 (1.16–6.30)	1.63 (0.56–4.74)
Pre-eclampsia^f^	14 (14.7)	9 (3.2)	23 (6.1)	5.20 (2.17–12.27)	5.29 (2.19–12.77)	7.12 (2.56–19.83)
Cesarean section^g^	20 (37.0)	28 (17.6)	48 (22.5)	2.75 (1.39–5.47)	2.77 (1.39–5.51)	2.91 (1.31–6.42)
Postpartum hemorrhage^g^	3 (5.6)	4 (2.5)	7 (3.3)	2.28 (0.49–10.53)	2.28 (0.49–10.55)	2.72 (0.43–17.10)

BMI: body mass index; CI: confidence interval; IQR: interquartile range; OR: odds ratio.

Data are presented as *n* (%) and median (IQR). Data were collected from registers.

a Data missing for 44 cases and 129 controls for univariate, age-adjusted, and multivariable OR.

b Data missing for four cases and three controls.

c ICD code E10*-E15*.

d ICD code O24*.

e ICD code O10-O13.

f Includes eclampsia.

g Analysis includes only women with postpartum ischemic stroke.

**Table 3.** Comparison of the number of risk factors in the ischemic stroke cases and controls.

Variable	Cases	Controls	All	Unadjusted OR (95% CI)	Age-adjusted OR (95% CI)
	*n* = 95	*n* = 280	*n* = 375		
At least one traditional risk factor^a,b,c^	31 (32.6)	55 (19.6)	86 (22.9)	2.12 (1.17–2.86)	2.15 (1.17–3.93)
At least one pregnancy-related risk factor^d^	30 (31.6)	44 (15.7)	74 (19.7)	2.48 (1.44–4.25)	2.53 (1.46–4.37)
Traditional and pregnancy-related risk factors^a,b^					
⩾1 risk factor	48 (50.5)	84 (30.0)	132 (35.2)	2.38 (1.48–3.84)	2.38 (1.48–3.84)
⩾2 risk factors	18 (18.9)	20 (7.1)	38 (10.1)	3.04 (1.53–6.03)	3.05 (1.53–6.06)
⩾3 risk factors	8 (8.4)	5 (1.8)	13 (3.5)	5.06 (1.61–15.86)	5.06 (1.61–15.90)
⩾4–5 risk factors	3 (3.2)	1 (0.36)	4 (1.1)	9.10 (0.94–88.54)	14.21 (1.12–180.48)

CI: confidence interval; OR: odds ratio.

Data are presented as *n* (%). Data were collected from registers.

a Smoking data missing for four cases and three controls.

b BMI data missing for 44 cases and 129 controls.

c Smoking, obesity, chronic hypertension, hypercholesterolemia, migraine, and/or diabetes mellitus.

d Gestational hypertension, pre-eclampsia, eclampsia, and/or diabetes during pregnancy.

The IS patients more frequently had at least one traditional and/or pregnancy-related stroke risk factor compared to controls (50.5% vs 30.0%, OR 2.38, 95% CI 1.48–3.84). The risk of IS increased with the number of risk factors: Having at least two risk factors (OR 3.04, 95% CI 1.53–6.03), three risk factors (OR 5.06, 95% CI 1.61–15.86), or four to five risk factors (age-adjusted OR 14.21, 95% 1.12–180.48) were more common in IS patients than in the controls. The maximum number of risk factors per person was five in both the cases and the controls.

IS patients with negative diagnostic evaluation also had more commonly traditional and/or pregnancy-related stroke risk factors compared to controls (47.5% vs 25.6%, OR 2.62, 95% CI 1.24–5.54) (Supplemental Table 4).

### IS incidence by age, pregnancy duration, and TOAST classification

The incidence of IS during 1987–2016 was 5.5 (95% CI 4.5–6.6) per 100.000 deliveries: 0.7 (95% CI 0.04–1.2) for cardioembolism, 0.11 (95% CI 0.02–0.36) for small vessel disease, 1.5 (95% CI 1.0–2.2) for other determined causes, and 3.2 (95% CI 2.4–4.0) for undetermined causes.

**Figure 2.**
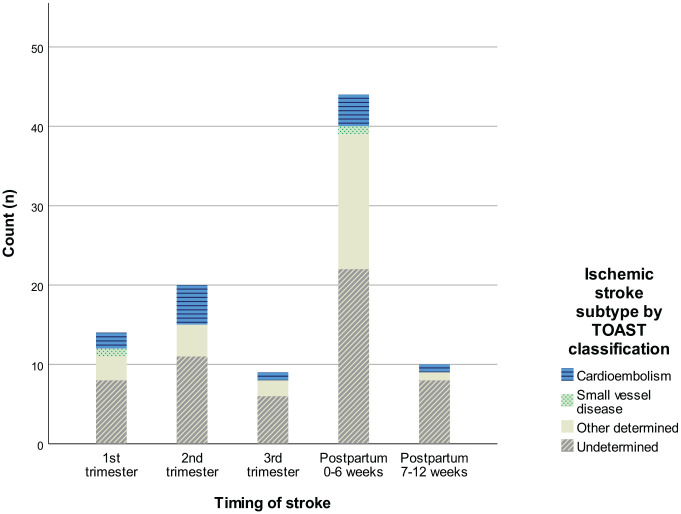
Distribution of ischemic stroke cases by gestational age and TOAST classification.

Of all the ISs, 14.4% presented in the first trimester, 20.6% in the second trimester, 9.3% in the third trimester, 45.4% within 6 weeks following delivery, and 10.3% during the late postpartum period (7–12 weeks after delivery) (Figure 2). The occurrence of ISs did not vary between the TOAST classes.

IS incidence by age and TOAST classes are presented in Supplemental Figure 1 and by 5-year time periods and TOAST classes in Supplemental Figure 2.

## Discussion

To our knowledge, this is the largest study cohort for IS in pregnancy and puerperium, in which all IS diagnoses were verified from patient records. The most common etiology for IS per TOAST classification was undetermined (57%), followed by other determined etiology (28%), cardioembolism (13%), and small-vessel occlusion (2%). Among these IS patients, two-thirds had traditional and one-third had pregnancy-related risk factors. Significant risk factors for pregnancy-related IS were pre-eclampsia or eclampsia, gestational hypertension, and migraine. IS patients had more frequently traditional and pregnancy-related stroke risk factors than healthy controls, and the risk of IS multiplied with the number of risk factors. Most ISs occurred within 6 weeks following delivery.

The most common etiologies of pregnancy-related IS found in other studies were cardiac embolism,^3,10,14,19^ coagulopathy,^3,13,19^ reversible cerebral vasoconstriction syndrome,^13^ and eclampsia.^5,14^ The proportion of undetermined etiology was similar (46%) in one study^3^ but smaller in others (10%–20%).^5,10,13,19^ However, other studies of pregnancy-related IS were not using TOAST classification, and the definitions of the etiologies were diverse. For example, we defined eclampsia as a risk factor of IS instead of an etiology. In 15–49 years old Finnish women, IS etiologies per TOAST classification were quite similar, since they had undetermined in 31%, other determined in 28%, cardioembolism in 20%, small-vessel disease in 11%, and large-artery atherosclerosis in 5%.^20^

Of those IS patients with undetermined etiology, 75% had a comprehensive, but negative diagnostic evaluation, and 24% had incomplete investigations. There were similar results in one study of undetermined ISs, where 77% had negative evaluation, and 16% had incomplete diagnostics.^20^ Inadequate diagnostics may be explained by a lack of diagnostic methods and protocols, since most of these strokes occurred before 1997. Comprehensively, but negatively, evaluated cases accounted for 42% of all ISs in our cohort, making it the largest group. However, this group had more risk factors than healthy controls and similar risk factors as other IS patients, except for having fewer HDP. ESUS criteria was met in 24% of negatively evaluated patients.

Pre-eclampsia or eclampsia was associated with five-fold, gestational hypertension was associated with three-fold, and migraine with a 37-fold risk of IS. A third of IS patients had HDP, and in general, pregnancy-related risk factors were associated with a 2.5-fold higher risk of IS. As much as 35% of our IS patients had migraine. One study reported that 25% of the increased risk of maternal stroke associated with migraine was mediated by HDP, which might also affect our results.^21^ U.S. studies have reported that pregnant patients had less traditional risk factors and were younger than non-pregnant IS patients.^11,12^ However, in our cohort, having at least one traditional risk factor was associated with a two-fold higher risk of IS and the effect of all risk factors appeared to be cumulative. Smoking, obesity, hypercholesterolemia, and chronic hypertension showed nonsignificant associations with IS, which might be affected by the small cohort size.

Our group previously reported that the incidence of pregnancy-related IS has risen in the past 30 years and with maternal age.^2^ There was lower incidence in our study, 5.5/100,000 deliveries, than reported in Asian, French, and U.S. studies with 13–21/100,000 deliveries.^3,5,14,19^ Overall, the estimates on incidence are largely variable due to different study methods and populations.^1^ The patient record validation excluded majority of potential cases identified from registers, and abortions before 22 weeks of gestation were excluded, which might affect our results. Also, Finland has nationwide and free-of-charge maternity care, which may improve prevention of stroke risk factors.

There are limitations in this study, such as a rather small sample size. We excluded pregnancies that resulted in abortion before 22 weeks of gestation, which could lead to underestimating ISs occurring in early pregnancy. The ethnical composition of the Finnish population is fairly homogenic, so our results may not be applicable to other ethnical groups. Risk factor data in case-control analyses were searched only from the registers, and there may be detection bias in diagnosing risk factors in IS cases compared to controls resulting in overestimation of their effect size. Large confidence intervals for migraine as risk factor suggest low degree of precision and warrant furth verification, preferably prospective studies. Furthermore, there are limitations to the TOAST classification. Definitions on subtypes rely on opinion and can cause inter-observer variability.^16,22^ The amount of cases with undetermined etiology may be overestimated due to incomplete diagnostics. TOAST classification does not specify the exact methods required for diagnostic evaluation, which can cause heterogeneity.^16^ It should be noted, that criteria for minimum diagnostic assessment by TOAST classification and ESUS were different. Finally, our results can be affected by changes in the diagnostic methods of IS due to retrospective data collection during a long time. Especially the diagnostics of PFOs and ASDs might be less precise in earlier years of the study period.

## Conclusions

We reported the etiology and risk factors of IS in pregnancy and puerperium in a population-based, nationwide, retrospective study. The strengths of this study are a long sampling period, its nationwide nature, comprehensive search strategy, and patient record validation. Pregnancy-related IS has become more common in recent decades and most frequently occurs in early postpartum. Half of the ISs had undetermined etiology, most of them despite excessive diagnostic evaluation. Also, cardioembolism and certain specified causes, such as arterial dissection, were frequent. Traditional and pregnancy-related stroke risk factors are associated with pregnancy-related IS, especially migraine, pre-eclampsia or eclampsia, and gestational hypertension. The risk of IS increases with the amount of risk factors. Adequate prevention and treatment of risk factors, such as hypertensive disorders, could decrease the morbidity and mortality caused by pregnancy-related IS. Follow-up and counseling are important especially in pregnant and postpartum women who have multiple identified risk factors for IS.

## Supplemental Material

sj-docx-1-eso-10.1177_23969873231170096 – Supplemental material for Etiology and risk factors of ischemic stroke during pregnancy and puerperium: A population-based study Click here for additional data file. Supplemental material, sj-docx-1-eso-10.1177_23969873231170096 for Etiology and risk factors of ischemic stroke during pregnancy and puerperium: A population-based study by Anna Richardt, Karoliina Aarnio, Aino Korhonen, Kirsi Rantanen, Liisa Verho, Hannele Laivuori, Mika Gissler, Minna Tikkanen and Petra Ijäs in European Stroke Journal

## References

[1] Swartz RH Cayley ML Foley N , et al. The incidence of pregnancy-related stroke: a systematic review and meta-analysis. Int J Stroke 2017; 12: 687–697. 28884652 10.1177/1747493017723271

[2] Karjalainen L Tikkanen M Rantanen K , et al. Stroke in pregnancy and puerperium: validated incidence trends with risk factor analysis in Finland 1987-2016. Neurology 2021; 96: e2564–e2575. 10.1212/WNL.0000000000011990 33827961

[3] Jaigobin C Silver FL . Stroke and pregnancy. Stroke 2000; 31: 2948–2951. 11108754 10.1161/01.str.31.12.2948

[4] Kamel H Navi BB Sriram N , et al. Risk of a thrombotic event after the 6-week postpartum period. N Engl J Med 2014; 370: 1307–1315. 24524551 10.1056/NEJMoa1311485 PMC4035479

[5] Sharshar T Lamy C Mas JL ; Stroke in Pregnancy Study Group. Incidence and causes of strokes associated with pregnancy and puerperium. A study in public hospitals of Ile de France. Stroke 1995; 26: 930–936. 7762040 10.1161/01.str.26.6.930

[6] Liu S Chan W-S Ray JG , et al. Stroke and cerebrovascular disease in pregnancy: incidence, temporal trends, and risk factors. Stroke 2019; 50: 13–20.

[7] Poorthuis MH Algra AM Algra A , et al. Female- and male-specific risk factors for stroke: a systematic review and meta-analysis. JAMA Neurol 2017; 74: 75–81. 27842176 10.1001/jamaneurol.2016.3482

[8] Ban L Sprigg N Abdul Sultan A , et al. Incidence of first stroke in pregnant and nonpregnant women of childbearing age: a population-based cohort study from England. J Am Heart Assoc 2017; 6: e004601. 10.1161/JAHA.116.004601 PMC5532991 28432074

[9] Martin A Lailler G Béjot Y , et al. Incidence and time trends of pregnancy-related stroke between 2010 and 2018: the nationwide conception study. Neurology 2022; 99: e1598–e1608. 10.1212/WNL.0000000000200944 PMC9559943 36038274

[10] Khan M Wasay M Menon B , et al. Pregnancy and puerperium-related strokes in Asian women. J Stroke Cerebrovasc Dis 2013; 22: 1393–1398. 23751156 10.1016/j.jstrokecerebrovasdis.2013.04.024

[11] Kittner SJ Stern BJ Feeser BR , et al. Pregnancy and the risk of stroke. N Engl J Med 1996; 335: 768–774. 8703181 10.1056/NEJM199609123351102 PMC1479545

[12] Leffert LR Clancy CR Bateman BT , et al. Treatment patterns and short-term outcomes in ischemic stroke in pregnancy or postpartum period. Am J Obstet Gynecol 2016; 214: 723.e1–723.e11. 10.1016/j.ajog.2015.12.016 26709084

[13] Yoshida K Takahashi JC Takenobu Y , et al. Strokes associated with pregnancy and puerperium: a nationwide study by the Japan Stroke Society. Stroke 2017; 48: 276–282. 28028148 10.1161/STROKEAHA.116.014406

[14] Skidmore FM Williams LS Fradkin KD , et al. Presentation, etiology, and outcome of stroke in pregnancy and puerperium. J Stroke Cerebrovasc Dis 2001; 10: 1–10. 17903792 10.1053/jscd.2001.20977

[15] Vandenbroucke JP von Elm E Altman DG , et al. Strengthening the Reporting of Observational Studies in Epidemiology (STROBE): explanation and elaboration. Int J Surg 2014; 12: 1500–1524. 25046751 10.1016/j.ijsu.2014.07.014

[16] Adams H Biller J . Classification of subtypes of ischemic stroke: history of the trial of Org 10 172 in acute stroke treatment classification. Stroke 2015; 46: e114–e117. 10.1161/STROKEAHA.114.007773 25813192

[17] van Swieten JC Koudstaal PJ Visser MC , et al. Interobserver agreement for the assessment of handicap in stroke patients. Stroke 1988; 19: 604–607. 3363593 10.1161/01.str.19.5.604

[18] Hart RG Diener H-C Coutts SB , et al. Embolic strokes of undetermined source: the case for a new clinical construct. Lancet Neurol 2014; 13: 429–438. 24646875 10.1016/S1474-4422(13)70310-7

[19] Jeng J-S Tang S-C Yip P-K . Incidence and etiologies of stroke during pregnancy and puerperium as evidenced in Taiwanese women. Cerebrovasc Dis 2004; 18: 290–295. 15331875 10.1159/000080354

[20] Putaala J Metso AJ Metso TM , et al. Analysis of 1008 consecutive patients aged 15 to 49 with first-ever ischemic stroke: the Helsinki young stroke registry. Stroke 2009; 40: 1195–1203. 19246709 10.1161/STROKEAHA.108.529883

[21] Bandoli G Baer RJ Gano D , et al. Migraines during pregnancy and the risk of maternal stroke. JAMA Neurol 2020; 77: 1177–1179. 32478828 10.1001/jamaneurol.2020.1435 PMC7265122

[22] Ay H Furie KL Singhal A , et al. An evidence-based causative classification system for acute ischemic stroke. Ann Neurol 2005; 58: 688–697. 16240340 10.1002/ana.20617

