# Supplementary material for: Etiology and risk factors of ischemic stroke during pregnancy and puerperium: A population-based study
Source: Eur Stroke J. 2023 Apr 24;8(2):475–82. doi: 10.1177/23969873231170096 (PMC10334185; doi:10.1177/23969873231170096)
Supplement: sj-docx-1-eso-10.1177_23969873231170096 – Supplemental material for Etiology and risk factors of ischemic stroke during pregnancy and puerperium: A population-based study [file sj-docx-1-eso-10.1177_23969873231170096.docx]

**Etiology and Risk Factors of Ischemic Stroke During Pregnancy and Puerperium - A Population-based Study**

Richardt Anna, M.D., Aarnio Karoliina, M.D., PhD., Korhonen Aino, Bc.M., Rantanen Kirsi, M.D., Verho Liisa, M.D., Laivuori Hannele, M.D., PhD., Gissler Mika, PhD., M.Soc.Sc, Tikkanen Minna, M.D., Ph.D., Petra Ijäs, M.D., Ph.D., M.Sc.

SUPPLEMENTAL MATERIALS

**Supplemental Methods**

**Supplemental Table 1.** Diagnostic Investigations in Ischemic Stroke Patients

**Supplemental Table 2.** Causes of Ischemic Strokes per TOAST Classification

**Supplemental Table 3.** Comparison of the IS cases with negative evaluation to other IS cases

**Supplemental Table 4.** Risk Factors for the IS cases with negative evaluation and Controls

**Supplemental Figure 1.** The Incidence of Ischemic Stroke by Age Groups and TOAST classes

**Supplemental Figure 2.** The Incidence of Ischemic Stroke by 5-year Time Periods and TOAST classes

**Strobe statement**

Supplemental Methods

**Register Data**

Women diagnosed with ischemic stroke (IS) during pregnancy or postpartum in Finland from 1987 to 2016 were identified by linking national healthcare registers; Medical Birth Register (MBR) ^1^, Register of Induced Abortions, Hospital Discharge Register/Care Register for Health Care (HDR) ^2^, and the Cause-of-Death register. The Medical Birth Register includes data on live births and on stillbirths of fetuses with a birth weight of at least 500 g or with a gestational age of at least 22 weeks, as well as baseline characteristics of the mother, and data on healthcare, pregnancy, delivery, and outcome of the newborn. Hospital Discharge Register (1969-1993) and the Care Register for Health Care (since 1994) include data on patients discharged from inpatient care in primary care, rehabilitation centers and hospitals. The Causes-of-Death Register includes Finnish residents' death certificates from 1936 onwards. The MBR and HDR are maintained by the Finnish Institute for Health and Welfare and reporting is obligatory for all healthcare facilities in Finland.^1,2^ The validity of MBR and HDR are estimated to be good or satisfactory.^3,4^

The HDR, MBR and Cause-of-Death registers were searched from 1987 to 2016 with disease or procedure codes indicating IS or its treatment in HDR up to 9 months (270 days) before or up to 3 months (90 days) after the delivery date in MBR. The data were collected retrospectively until the end of year 2016. The search strategy was purposefully comprehensive to identify all possible cases. The disease and procedure codes were collected from the International Statistical Classification of Diseases and Related Health Problems (ICD) and NOMESCO Classification of Surgical Procedures (NCSP). The diagnostic codes used to identify patients with IS during pregnancy or puerperium were 4330A, 4331A, 4339A, 4340A, 4341A, 4349A in ICD-9 and I63 in ICD-10. The procedure codes applied were AAL10, TPX22, PA2AT, PA2BT, PA2CT, PA2ST, PA2VT, PAE12, PAE14, PAF12, PAF13, PAF14, PAF15, PA2YT, PA6YT, PA7XT, PA7YT, PA8YT, PAQ12, PAQ14, PA4AT, PA4YT, PA6AT, PAF12, and PAF14.

The data of traditional and pregnancy-related stroke risk factors were collected from the MBR and HDR. Data in the MBR are supplemented by healthcare professionals during pregnancy in antenatal clinic visits and until 7 days after delivery in maternity hospitals. BMI data have been collected in the MBR since 2004. The HDR was searched with ICD diagnostic codes corresponding to stroke risk factors registered during pregnancy and until 3 months after delivery. The diagnostic codes used to identify stroke risk factors were 250* in ICD-9 and E10*–E15* in ICD-10 for diabetes mellitus, 346* in ICD-9 and G43* in ICD-10 for migraine, 401*–404*, 6420*–6423* and 6429* in ICD-9 and I10*–I13*, O10*–O13*, and O16* in ICD-10 for chronic hypertension, 6424*–6427* in ICD-9 and O14*–O15* in ICD-10 for pre-eclampsia or eclampsia, and 6480A and 6488A in ICD-9 and O24* in ICD-10 for diabetes during pregnancy.

**Definitions**

The definition of IS used in this study was: brain cell death attributable to ischemia, based on pathological, imaging, or other objective evidence of cerebral focal ischemic injury in a defined vascular distribution; or clinical evidence of cerebral focal ischemic injury based on symptoms persisting ≥24 hours or until death, and other etiologies excluded.^5^ Spinal cord or retinal focal ischemic injury were not included in this study. Patients who did not have pregnancy- or puerperium-associated ISs were excluded, and the reasons of exclusion were recorded. Also, patients with transient ischemic attacks and infarctions caused by cerebral venous thrombosis were excluded.

Pregnancy was defined as starting from conception and ending at delivery, and postpartum period lasting until 12 weeks (84 days) after delivery. Delivery was defined as delivery of a newborn with a birth weight over 500 g or with gestational weeks over 22+0.

Pre-eclampsia was defined as blood pressure >140/90 or elevated >30/15 from baseline and >0.5 g/day proteinuria. Eclampsia was defined as pre-eclampsia combined with seizures. Smoking was defined as daily or occasional smoking during pregnancy, including those who quit before 12 weeks of gestation. Migrainous infarction was defined as IS in a relevant area in neuroimaging, when patient has a typical migraine attack and aura symptom(s) persisting for >60 minutes.^6^ Traditional risk factors included smoking, obesity (BMI ≥30 kg/m2), chronic hypertension, hypercholesterolemia, migraine and diabetes mellitus. Pregnancy-related risk factors included gestational diabetes or diabetes during pregnancy, gestational hypertension, pre-eclampsia, eclampsia and hemolysis, elevated liver enzymes, low platelets syndrome (HELLP).

We defined the etiologies of IS using the Trial of ORG 10172 in Acute Stroke Treatment (TOAST) criteria, which included large-artery atherosclerosis, cardioembolism, small-vessel occlusion, stroke of other determined etiology, and stroke of undetermined etiology.^7^ Stroke of undetermined etiology had three subclasses: two or more causes identified, negative evaluation, or incomplete evaluation.^7^ The evaluation was considered incomplete if brain imaging (computed tomography (CT) or magnetic resonance imaging (MRI)), vessel imaging (CT or MR angiography, carotid ultrasound, or DSA), echocardiogram (transthoracic or transesophageal with bubble test with contrast agent at the discretion of cardiologist), electrocardiogram or basic laboratory assessments (blood count and platelets, prothrombin time or INR, PTT, serum electrolytes, blood glucose, hepatic and renal chemical analyses) were not investigated. Laboratory examinations for the prothrombotic state included FV-D, FII-D, TT, AT3, protein C, protein S, lupus anticoagulant, cardiolipin immunoglobulins, beta-2-glycoprotein, and thrombin time.

Embolic stroke of undetermined source (ESUS) was defined as a non-lacunar stroke detected by CT or MRI, in the absence of extra- or intracranial atherosclerosis causing ≥50% luminal stenosis, major risk cardioembolic source and other specific cause of stroke, when minimum diagnostic assessment (defined as brain CT or MRI, imaging of the extra- and intracranial arteries, ECG, echocardiography and Holter) was done.^8^

**Data Collected from Medical Records**

Medical records were acquired from healthcare facilities where the disease or procedure codes indicating IS during pregnancy or puerperium were coded. We acquired records from the specialties of neurology, obstetrics and gynecology, neurosurgery, emergency, internal medicine, radiology, and laboratory medicine. More patient records were ordered if needed after the first review of them. In Finland, electronic patient records have been used nationwide at the latest in 2007. Before this, patient records have been systematically written and archived locally in all Finnish hospitals.

The diagnosis of IS and its temporal connection to pregnancy were verified from the medical records by stroke neurologists. For the IS cases, the following clinical data were collected from hospital records: demographics (age, parity, ethnicity, smoking, substance abuse, migraine, diabetes, hypertension, cardiac disease, hyperlipidemia, prothrombotic disorders, previous thromboembolic events, other significant diseases, medications, prior infections and trauma, family history of stroke), subtype of stroke, timing of stroke onset, imaging results, other examinations (electrocardiography, echocardiography, laboratory tests), treatment (acute treatment, secondary prevention), outcome (mortality, functional outcome prior event, at discharge, after three months and one year, length of hospital stay, discharge destination), obstetric data (type of delivery, delivery complications), pregnancy complications (pre-eclampsia, eclampsia, gestational diabetes, other hypertensive disorders), subsequent pregnancies, recurrent strokes, cardiovascular diseases and other morbidities during follow-up (until last medical record or until 2016). Data from medical records was collected until end of year 2016.

**Statistics**

The incidence of IS was reported per 100,000 deliveries. Incidence rates were calculated for 5-year maternal age groups (<20, 20-24, 25-29, 30-34, 35-39, 40-44, ≥45) and 5-year time periods (1987–1991, 1992–1996, 1997–2001, 2002–2006, 2007–2011, 2012–2016). The incidence per pregnancy trimesters (conception to 12+0 weeks, 12+1 to 28+0 weeks, over 28+1 weeks) and by postpartum week, and per TOAST classes.

**References**

1. THL. Medical Birth Register. Accessed June 5, 2020. DOI: thl.fi/en/web/thlfi-en/statis tics/information-on-statistics/register-descriptions/newborns.

2. THL. Care register for health care. Accessed June 5, 2020. DOI: thl.fi/en/web/thlfi-en/statistics/information-on-statistics/register-descriptions/care-register-for-healthcare.

3. Sund R. Quality of the Finnish Hospital Discharge Register: a systematic review. *Scand J Public Health* 2012; 40: 505-515.

4. Gissler M, Teperi J, Hemminki E, et al. Data quality after restructuring a national medical registry. *Scand J Soc Med* 1995; 23: 75-80.

5. Sacco RL, Kasner SE, Broderick JP, et al. An updated definition of stroke for the 21st century: a statement for healthcare professionals from the American Heart Association/American Stroke Association. *Stroke* 2013; 44: 2064-2089.

6. Society HCCotIH. The international classification of headache disorders. *Cephalalgia* 2013; 33: 629-808.

7. Adams H and Biller J. Classification of Subtypes of Ischemic Stroke: History of the Trial of Org 10 172 in Acute Stroke Treatment Classification. *Stroke* 2015; 46: e114-e117.

8. Hart RG, Diener H-C, Coutts SB, et al. Embolic strokes of undetermined source: the case for a new clinical construct. *The Lancet Neurology* 2014; 13: 429-438.

**Supplemental Table 1. Diagnostic Investigations in Ischemic Stroke Patients**

| Investigations | IS cases, n (%) |
| --- | --- |
| Brain native CT or MRI | 97 (100) |
| Brain native CT | 59 (60.8) |
| Brain MRI | 78 (80.4) |
| Brain native CT and MRI | 40 (41.2) |
| Brain perfusion CT | 3 (3.1) |
| Vessel imaging | 82 (84.5) |
| CTA | 20 (20.6) |
| MRA | 51 (52.6) |
| Carotid ultrasound | 13 (13.4) |
| DSA | 17 (17.5) |
| Holter monitoring | 29 (29.9) |
| Echocardiogram (TTE or TEE) | 79 (81.4) |
| TCD bubble test or the dye dilution method | 5 (5.2) |
| Lumbal puncture | 13 (13.4) |
| Laboratory examinations for prothrombotic state | 64 (66.0) |

Abbreviations: CT = computed tomography; CTA = computed tomography angiography; DSA = digital subtraction angiography; MRI = magnetic resonance imaging; MRA = magnetic resonance angiography; TCD = transcranial doppler; TEE = transesophageal echocardiogram; TTE = transthoracic echocardiogram.

**Supplemental Table 2. Causes of Ischemic Strokes per TOAST Classification**

| TOAST class |  | n (%/TOAST class) |
| --- | --- | --- |
| Cardiac embolism (n=13) | ASD | 5 (38.5) |
|  | PFO | 4 (30.8) |
|  | Atrial fibrillation | 1 (7.7) |
|  | Fallot tetralogy and heart dysfunction | 1 (7.7) |
|  | Left atrial thrombus | 1 (7.7) |
|  | Vegetations on mitral prosthetic valve | 1 (7.7) |
| Stroke of other determined etiology (n=27) | Dissection of cervical or intracranial artery | 11 (40.7) |
|  | HELLP with associated DIC | 3 (11.1) |
|  | Factor V Leiden mutation | 3 (11.1) |
|  | Antiphospholipide syndrome | 3 (11.1) |
|  | Hyperstimulation syndrome | 2 (7.4) |
|  | Hypovolemia | 1 (3.7) |
|  | Air embolism | 1 (3.7) |
|  | Moyamoya disease | 1 (3.7) |
|  | PRES | 1 (3.7) |
|  | Migraine | 1 (3.7) |
| Stroke of undetermined etiology (n=55) | Two or more identified causes  - vasculitis and atrial fibrillation | 1 (1.8) |
|  | Negative evaluation | 41 (74.6) |
|  | Incomplete evaluation | 13 (23.6) |

Abbreviations: ASD = atrial septal defect; PFO = patent foramen ovale; HELLP = hemolysis, elevated liver enzymes, low platelets; PFO = patent foramen ovale; PRES = posterior reversible encephalopathy syndrome; TOAST = Trial of Org 10172 in Acute Stroke Treatment.

**Supplemental Table 3. Comparison of the IS cases with negative evaluation to other IS cases**

| Etiology | Negative evaluation IS cases n=41 | Other IS cases n=56 | All n=97 | Unadjusted OR (95 % CI) |
| --- | --- | --- | --- | --- |
| Age (years), median ± IQR | 29.9 ± 7.5 | 31.4 ± 9.1 | 30.7 ± 8.3 | 0.974 (0.90–1.05) |
| Parity, median ± IQR | 1 ± 2 | 2 ± 3 | 2 ± 3 | 0.87 (0.70–1.08) |
| BMI (kg/m^2^)^a^, median ± IQR | 21.9 ± 5.4 | 23.0 ± 2.6 | 22.7 ± 5.6 | 0.947 (0.85–1.06) |
| Traditional risk factors^a,b^ | 27 (65.9) | 38 (67.9) | 65 (67.0) | 0.914 (0.39–2.15) |
| Obesity (BMI≥30 kg/m^2^)^a^ | 2 (4.9) | 6 (10.7) | 8 (8.2) | 0.427 (0.082–2.24) |
| Smoking^b^ | 6 (14.6) | 9 (16.1) | 15 (15.5) | 0.895 (0.29-2.75) |
| Chronic hypertension | 2 (4.9) | 5 (8.9) | 7 (7.2) | 0.523 (0.96–2.84) |
| Hypercholesterolemia | 17 (41.5) | 18 (32.1) | 35 (36.1) | 1.495 (0.29–1.54) |
| Migraine | 19 (36.6) | 15 (33.9) | 34 (35.1) | 1.123 (0.38–2.07) |
| Migraine with aura | 14 (34.1) | 14 (25.9) | 28 (29.5) | 1.481 (0.61–3.60) |
| Diabetes, type I or II | 0 | 0 | 0 | N/A |
| Pregnancy related risk factors | 10 (24.4) | 24 (42.9) | 34 (35.1) | 0.430 (0.18–1.05) |
| Gestational diabetes | 6 (14.6) | 10 (17.9) | 16 (16.5) | 0.789 (0.26–2.38) |
| Gestational hypertension | 3 (7.3) | 7 (12.5) | 10 (10.4) | 0.553 (0.13-2.28) |
| Pre-eclampsia^c^ | 4 (9.7) | 17 (30.4) | 21 (21.6) | 0.237 (0.063–0.89)* |
| Hypertensive disorders of pregnancy^d^ | 7 (17.1) | 21 (37.5) | 28 (28.9) | 0.343 (0.13-0.91)* |
| At least one traditional or pregnancy-related risk factor^a,b^ | 30 (73.2) | 48 (85.7) | 78 (80.4) | 0.455 (0.16-1.26) |
| Maternal mortality | 0 | 5 (8.9) | N/A | N/A |
| Good functional outcome (mRS 0-2) at discharge | 35 (85.4) | 36 (64.3) | 71 (73.2) | 3.241 (1.16-9.02)* |
| Good functional outcome (mRS 0-2) at 3 months | 37 (90.2) | 44 (78.6) | 81 (83.5) | 2.523 (0.75-8.49) |

Data are presented as n (%) and median ± IQR. Abbreviations: BMI = body mass index; HELLP = hemolysis, elevated liver enzymes, low platelets syndrome; IQR = interquartile range; IS = ischemic stroke; mRS = Modified Rankin scale. ^a^BMI data missing in 44 cases. ^b^Smoking data missing in 4 cases. ^c^Includes eclampsia and HELLP. ^d^Pre-eclampsia, eclampsia, HELLP, gestational hypertension, and/or chronic hypertension.

**Supplemental Table 4. Risk Factors for the IS cases with negative evaluation and Controls**

| Variable | Negative evaluation IS cases n=40^a^ | Controls n=117^a^ | All n=157 | Unadjusted OR (95 % CI) |
| --- | --- | --- | --- | --- |
| Age (years), median ± IQR | 29.0 ±7.5 | 29.0 ± 8.0 | 29.0 ± 8.0 | 1.001 (0.93–1.078) |
| Parity, median ± IQR | 1 ± 2 | 1 ± 2 | 1 ± 2 | 0.896 (0.66–1.21) |
| BMI (kg/m^2^)^b^, median ± IQR | 22.0 ± 5.8 | 23.1 ± 4.7 | 22.7 ± 5.0 | 1.000 (0.91-1.10) |
| Traditional risk factors^b,c^ | 13 (32.5) | 19 (16.2) | 32 (20.4) | 2.480 (1.01-6.08)* |
| Obesity (BMI≥30 kg/m^2^)^b^ | 2 (8.0) | 6 (8.2) | 8 (5.1) | 0.971 (0.18-5.15) |
| Smoking^c^ | 6 (15.3) | 16 (13.9) | 22 (14.0) | 1.125 (0.41–3.11) |
| Chronic hypertension | 1 (2.5) | 0 | 1 (0.6) | N/A |
| Hypercholesterolemia | 1 (2.5) | 1 (0.9) | 2 (1.3) | 2.97 (0.18–48.69) |
| Migraine | 7 (17.5) | 0 | 7 (4.5) | N/A |
| Diabetes mellitus^d^ | 0 | 0 | 0 | N/A |
| Pregnancy-related risk factors | 10 (25.0) | 13 (11.1) | 23 (14.6) | 2.667 (1.06-6.69)* |
| Diabetes during pregnancy^e^ | 6 (15.0) | 8 (6.8) | 14 (8.9) | 2.400 (0.78–7.42) |
| Gestational hypertension | 4 (10.0) | 5 (4.3) | 9 (5.7) | 2.489 (0.63–9.77) |
| Pre-eclampsia^f^ | 4 (10.0) | 1 (0.9) | 5 (3.2) | 12.890 (1.40–119.02)* |
| At least one traditional or pregnancy-related risk factor^b,c^ | 19 (47.5) | 30 (25.6) | 49 (31.2) | 2.624 (1.24-5.54)* |

Data are presented as n (%) and median (IQR). Abbreviations: BMI = body mass index; CI = confidence interval; IQR = interquartile range; IS = ischemic stroke; OR = odds ratio. ^a^1 case and 3 controls were not included in the analysis due to lack of adequate register data. ^b^BMI data missing in 15 cases and 44 controls. ^c^Smoking data missing in 1 case and 2 controls. ^d^ICD code E10*-E15*. ^e^ICD code used O24*. ^f^Includes eclampsia.

**Supplemental Figure 1. The Incidence of Ischemic Stroke by Age Groups and TOAST classes**

**Supplemental Figure 2. The Incidence of Ischemic Stroke by 5-year Time Periods and TOAST classes**

**STROBE Statement—checklist of items that should be included in reports of observational studies**

|  | Item No. | Recommendation | Page  No. | Relevant text from manuscript |
| --- | --- | --- | --- | --- |
| **Title and abstract** | 1 | (*a*) Indicate the study’s design with a commonly used term in the title or the abstract | 1 |  |
|  |  | (*b*) Provide in the abstract an informative and balanced summary of what was done and what was found | 2 |  |
| Introduction | | | |  |
| Background/rationale | 2 | Explain the scientific background and rationale for the investigation being reported | 3 |  |
| Objectives | 3 | State specific objectives, including any prespecified hypotheses | 3 |  |
| Methods | | | |  |
| Study design | 4 | Present key elements of study design early in the paper | 4 |  |
| Setting | 5 | Describe the setting, locations, and relevant dates, including periods of recruitment, exposure, follow-up, and data collection | 4 |  |
| Participants | 6 | (*a*) *Cohort study*—Give the eligibility criteria, and the sources and methods of selection of participants. Describe methods of follow-up  *Case-control study*—Give the eligibility criteria, and the sources and methods of case ascertainment and control selection. Give the rationale for the choice of cases and controls  *Cross-sectional study*—Give the eligibility criteria, and the sources and methods of selection of participants | 4-5, Supplemental Methods |  |
|  |  | (*b*) *Cohort study*—For matched studies, give matching criteria and number of exposed and unexposed  *Case-control study*—For matched studies, give matching criteria and the number of controls per case | 5 |  |
| Variables | 7 | Clearly define all outcomes, exposures, predictors, potential confounders, and effect modifiers. Give diagnostic criteria, if applicable | 5 |  |
| Data sources/ measurement | 8* | For each variable of interest, give sources of data and details of methods of assessment (measurement). Describe comparability of assessment methods if there is more than one group | 4-5, Supplemental methods |  |
| Bias | 9 | Describe any efforts to address potential sources of bias | 4 |  |
| Study size | 10 | Explain how the study size was arrived at | 4, Figure 1 |  |

Continued on next page

| Quantitative variables | 11 | Explain how quantitative variables were handled in the analyses. If applicable, describe which groupings were chosen and why | 5-6 |  |
| --- | --- | --- | --- | --- |
| Statistical methods | 12 | (*a*) Describe all statistical methods, including those used to control for confounding | 5-6 |  |
|  |  | (*b*) Describe any methods used to examine subgroups and interactions |  |  |
|  |  | (*c*) Explain how missing data were addressed | Tables 1-3 |  |
|  |  | (*d*) *Cohort study*—If applicable, explain how loss to follow-up was addressed  *Case-control study*—If applicable, explain how matching of cases and controls was addressed  *Cross-sectional study*—If applicable, describe analytical methods taking account of sampling strategy |  |  |
|  |  | (*e*) Describe any sensitivity analyses |  |  |
| Results | | | | |
| Participants | 13* | (a) Report numbers of individuals at each stage of study—eg numbers potentially eligible, examined for eligibility, confirmed eligible, included in the study, completing follow-up, and analysed | Figure 1 |  |
|  |  | (b) Give reasons for non-participation at each stage | Figure 1 |  |
|  |  | (c) Consider use of a flow diagram | Figure 1 |  |
| Descriptive data | 14* | (a) Give characteristics of study participants (eg demographic, clinical, social) and information on exposures and potential confounders | Table 1 |  |
|  |  | (b) Indicate number of participants with missing data for each variable of interest | Tables 1-3 |  |
|  |  | (c) *Cohort study*—Summarise follow-up time (eg, average and total amount) |  |  |
| Outcome data | 15* | *Cohort study*—Report numbers of outcome events or summary measures over time | Table 1 |  |
|  |  | *Case-control study—*Report numbers in each exposure category, or summary measures of exposure | Tables 2-3 |  |
|  |  | *Cross-sectional study—*Report numbers of outcome events or summary measures |  |  |
| Main results | 16 | (*a*) Give unadjusted estimates and, if applicable, confounder-adjusted estimates and their precision (eg, 95% confidence interval). Make clear which confounders were adjusted for and why they were included | Tables 2-3 |  |
|  |  | (*b*) Report category boundaries when continuous variables were categorized | Figure 2, Supplemental Figures 1-2 |  |
|  |  | (*c*) If relevant, consider translating estimates of relative risk into absolute risk for a meaningful time period |  |  |

Continued on next page

| Other analyses | 17 | Report other analyses done—eg analyses of subgroups and interactions, and sensitivity analyses | Supplemental Tables 3-4 |  |
| --- | --- | --- | --- | --- |
| Discussion | | | | |
| Key results | 18 | Summarise key results with reference to study objectives | 13 |  |
| Limitations | 19 | Discuss limitations of the study, taking into account sources of potential bias or imprecision. Discuss both direction and magnitude of any potential bias | 15 |  |
| Interpretation | 20 | Give a cautious overall interpretation of results considering objectives, limitations, multiplicity of analyses, results from similar studies, and other relevant evidence | 13-15 |  |
| Generalisability | 21 | Discuss the generalisability (external validity) of the study results | 15 |  |
| Other information | |  | | |
| Funding | 22 | Give the source of funding and the role of the funders for the present study and, if applicable, for the original study on which the present article is based | Disclosures |  |

*Give information separately for cases and controls in case-control studies and, if applicable, for exposed and unexposed groups in cohort and cross-sectional studies.

**Note:** An Explanation and Elaboration article discusses each checklist item and gives methodological background and published examples of transparent reporting. The STROBE checklist is best used in conjunction with this article (freely available on the Web sites of PLoS Medicine at http://www.plosmedicine.org/, Annals of Internal Medicine at http://www.annals.org/, and Epidemiology at http://www.epidem.com/). Information on the STROBE Initiative is available at www.strobe-statement.org.
